# Managing a moral identity in debt advice conversations

**DOI:** 10.1111/bjso.12303

**Published:** 2018-12-05

**Authors:** Nicole Andelic, Clifford Stevenson, Aidan Feeney

**Affiliations:** ^1^ Queen's University Belfast UK; ^2^ Nottingham Trent University UK

**Keywords:** advice‐giving, conversation analysis, discursive psychology, institutional talk, personal debt, service‐use, stigma

## Abstract

Previous research has found that stigma can be a barrier to service use but there has been little work examining actual service encounters involving members of stigmatized groups. One such group are those with problematic or unmanageable debts. Providing advice to members of this group is likely to be particularly difficult due to the stigma associated with being in debt. Using conversation analysis and discursive psychology, this study examines 12 telephone advice conversations between debt advisors and individuals in debt. Both clients and advisors oriented to the negative moral implications of indebtedness and typically worked collaboratively to manage these issues. Clients often claimed a moral disposition as a way to disclaim any unwanted associations with debt, but could find it difficult to reconcile this with an insolvency agreement. Moreover, the institutional requirements of the interaction could disrupt the collaborative management of stigma and advisors could manage the subsequent resistance from clients in either client‐centred or institution‐centred ways. The findings suggest that the products offered by debt advice agencies, as well as the manner in which they are offered to clients, can either help or hinder debtors negotiate the stigma‐related barriers to service engagement.

## Background

In contemporary societies, people can typically access a range of advice, services, and interventions to help them cope with the challenges of their everyday lives. Paradoxically, the people who most need to access these services are often the least likely to do so. Indeed, research has found that many people who are in need of help in the form of food banks (Fong, Wright, & Wimer, 2016), community services (Stevenson, McNamara, & Muldoon, 2014), debt advice (Goode & Waring, 2011), and mental health services (Livingston & Boyd, 2010) do not avail of that help.

There are a range of possible explanations for this paradox, but one important factor is the stigma which people associate with being poor, having unmanageable debts or having mental health problems. There is a large literature on the potential consequences of having a stigmatized identity: social exclusion (Link & Phelan, 2001); lower self‐esteem and poor mental health (Major & O'Brien, 2005); and impaired cognitive performance (Nguyen & Ryan, 2008) and all of these factors demonstrably act as barriers to accessing services. Traditionally, stigma is understood as a socially constructed attribute which is considered a character defect or flaw, leading to negative experiences for the stigmatized (Major & O'Brien, 2005). What is less well understood is the role that stigma plays in the unfolding of interactions whereby interlocutors must attend to delicate issues of moral accountability while accomplishing their interactive goals. This is potentially extremely relevant to service use encounters where stigmatized individuals have typically overcome initial cognitive and social barriers to requesting help, but then face the challenge of a morally charged interaction with a service provider. Although we do not examine stigma directly, in this study, we examine a specific type of particularly difficult service encounter: that between individuals with very serious problem debts and debt advisors seeking to help them choose a debt resolution mechanism. Studying such encounters will help us to understand the nature of the difficulties presented by potentially stigmatizing situations as well as the interaction strategies used by both parties to the encounter and their consequences.

### Stigma as barrier to service use

It is well established that stigma may hinder service use, especially mental health services (Ben‐Zeev, Corrigan, Britt, & Langford, 2012; Clement *et al*., 2015). Stigma has also been found to be a barrier to seeking care for HIV and other sexually transmitted diseases (Fortenberry *et al*., 2002; Kinsler, Wong, Sayles, Davis, & Cunningham, 2007), to mitigate against service use in disadvantaged communities (Stevenson *et al*., 2014; Warr, Davern, Mann, & Gunn, 2016), and racial stigma (Howarth, 2006) has serious consequences for service use (Campbell & McLean, 2002; McLean, Campbell, & Cornish, 2003).

One way that stigma works as a barrier to service use is due to negative associations with a particular service and the fear of being labelled or categorized as a service user. There are several studies which show how people avoid seeking help so as not to be labelled as mental health patients (Corrigan, 2004; Vogel, Wade, & Hackler, 2007). The fear of being included in the stigmatized category is also found in other instances of service use. For example, Fong *et al*. (2016) found that low‐income individuals distanced themselves from food banks, despite the great benefit food banks offer, partly due to undesirable characteristics associated with the people who queue for food. In all of these cases, services are used less than they might be because of stigma associated with being a service user.

Stigma can also lead people to disengage with services due to expectations of being treated negatively by service providers on the basis of a broader group membership. For example, Stevenson *et al*. (2014) examined the experience of being a community member of a socially disadvantaged neighbourhood. Perceptions of prejudice from service providers led community members to either distance themselves from service use, or to expect conflictual service use encounters. Thus, stigma can impede service use because potential users perceive service encounters to be a potential site of discrimination.

It is clear that many people who would benefit from utilizing services are not accessing them out of fear of stigma. However, while the current literature has examined the retrospective accounts of stigmatization provided by service providers and stigmatized individuals (Stevenson *et al*., 2014; Warr *et al*., 2016), it has focused less on investigating how stigma influences the service encounter itself.

In a rare example of research on debt advice conversations, Ekström, Lindström, and Karlsson (2013) found that talking about money is a delicate concern and that debtors presented themselves as responsible characters when organizing their ‘trouble‐tellings’. For example, callers made an effort to produce an account for why they were renegotiating their payment loans. They also demonstrated self‐awareness of their issues, emphasized the temporary nature of their payment issues, and outlined the steps they had already taken to solve their money problems. This suggests that debt advice conversations are potentially stigmatizing situations and conversations with service users who are concerned about being stigmatized are likely to be difficult. Adding to this research, in our study, we examined service encounters between debt advisors and people with problem debts.

### Specific barriers to seeking debt advice

Across England and Wales, 247 people are declared insolvent each day (The Money Charity, October 2016), suggesting that many people could avail of debt advice. In the United Kingdom, there are a range of services available for people who are struggling with debt including legal debt restructuring plans such as Individual Voluntary Arrangements (IVAs) and bankruptcy (The Insolvency Register, 2015). Estimates of the exact number of individuals with debt problems who have sought help vary, but one study found that only 8% of those who reported needing debt advice have sought it (Department for Business, Innovation & Skills, 2011). Thus, there appears to be a large group of people who may be suffering from the consequences of problem debt and need advice, but who have not taken any steps towards accessing help.

Barriers to seeking debt advice include lack of confidence (Goode & Waring, 2011), lack of knowledge (Goode & Waring, 2011; Pleasance, Buck, Balmer, & Williams, 2007) but perhaps most important of all, feelings of embarrassment and shame (Dearden, Goode, Whitfield, & Cox, 2010; Goode & Waring, 2011). Talking about financial difficulties is related to concerns about one's moral character (Ekström *et al*., 2013) and qualitative interviews found that people in debt perceived their own debt as evidence of lack of willpower or self‐control (Hayes, 2000; Keene, Cowan, & Baker, 2015). This self‐stigma associated with problem debt can lead to people hiding their debt from family members and isolating themselves for fear of peers finding out about their financial difficulties (Hayes, 2000; Thorne & Anderson, 2006). Thus, the stigma associated with being in debt in turn makes it less likely that people access freely available debt advice.

Ignoring debt may have severe consequences for one's mental and physical health. A large body of research shows an association between debt and poor well‐being (Brown, Taylor, & Price, 2005; Richardson, Elliott, & Roberts, 2013), suicidal ideation (Meltzer *et al*., 2011), increased rates of mental health disorder (Drentea & Reynolds, 2012), and poorer physical health (Drentea & Lavrakas, 2000). Mental and physical health problems are in turn likely to act as further barriers to accessing debt advice and may also make the debt advice conversation more difficult when it does occur.

### Advice conversations

Research on debt advice has focused mainly on the accessibility of advice and rates of successful outcomes after seeking advice (Orton, 2010; Pleasance *et al*., 2007) rather than analysing how debt advice conversations unfold in specific advice encounters. When examining interactions between service providers and stigmatized groups where identity management is relevant, it is useful to take advantage of an approach which examines conversations on a micro‐social level, such as discursive psychology (DP). Edwards (2005) suggests that there are two main features of DP; that language is situated and action‐oriented. This means that talk carries out an underlying action that people are skilfully picking up on, although they may not explicitly notice it. In addition to discursive psychology, the current study used elements of conversation analysis (CA) when conducting the analysis, which has been used to examine troubles‐talk in institutional settings previously (Ekström *et al*., 2013; Heritage & Lindström, 1998). Originally founded by Harvey Sacks (Heritage, 2005), CA examines the organization of talk in naturally occurring conversations to understand the performative action of words, phrases, and silences and how these are coordinated.

From a discursive psychology perspective, advice needs to be differentiated depending on whether it is given in a mundane or an institutional setting (Heritage & Sefi, 1992). Institutional talk is considered to have three particular characteristics that differentiate it from non‐institutional interactions: there are institutional identities with relevant goals determining the talk; there are constraints on the talk which occurs due to the setting; and there are specific inferences due to the context. Institutional advice‐giving is also asymmetric: medical consultations between physicians and patients are examples of institutional talk where one participant is established as the ‘expert’ in comparison with the other through interaction (Heritage, 2005; Maynard, 1991; Peräkylä, 1993). CA and DP have been used in research on a range of institutional advice contexts, including helplines (Butler, Potter, Danby, Emmison, & Hepburn, 2010; Emmison, Butler, & Danby, 2011; Hepburn, 2005; Potter & Hepburn, 2003), police interviews (Stokoe & Edwards, 2008), conversations between health visitors and first‐time mothers (Heritage & Lindström, 1998; Heritage & Sefi, 1992), pharmacists and patients (Pilnick, 2003), peer tutoring (Waring, 2005, 2007), and renegotiation of student loans (Ekström *et al*., 2013). However, although Ekström *et al*.'s (2013) study examined advice on paying student loans, to our knowledge problem debt advice has not been examined.

From the findings by Ekström *et al*. (2013), Hayes (2000), and Keene *et al*. (2015), we might expect that talk in debt advice conversations will manage identities to avoid the negative moral judgements associated with indebtedness. Debt advice conversations are likely to be problematic due to both the sensitive nature of the topic and the institutional constraints upon the conversation. The institutional goal for the advisor is to assess the debtors’ financial difficulties and advise appropriately, whereas debtors may have an additional goal of managing the accounts of their situations in order to avoid negative inferences about their moral character and behaviour. Sensitive topics, and a variety of means for handling them, have been uncovered in other discursive studies of service encounters such as between midwives and expectant mothers (Linnell & Bredmar, 1996), doctor–patient (Haakana, 2001) and client–counsellor interactions (Solberg, 2011). Given that none of these studies involved the participants being members of categories with negative associations, the challenges posed by the sensitive topic of indebtedness are likely to be greater.

Elsewhere, discursive studies demonstrate that examining service interactions can have practical implications for service providers. Such examinations can be used to identify specific problems which may occur (Potter & Hepburn, 2003), as well as motivating recommendations in the shape of interventions (Stokoe, 2014). Wiggins and Hepburn (2007) provide examples of how discursive research allows for the advisor to understand their own abilities and make changes to their current method of advice delivery. It is our hope that examining debt advice conversations from a discursive perspective would have a similar usefulness, for both the advisor and the advisee.

### The current study: Debt advice

In order to help us understand how service users and providers manage the sensitive issues around debt in service encounters, we examined how the conversations related to service use unfold from an interactional perspective. The particular conversations we studied were initial advice appointments between debt advisors and people with problem debts (hereafter called ‘clients’ in this study) at a financial advice organization, a private company which provides IVAs in the United Kingdom. The IVA is a formal debt resolution mechanism which allows individuals to pay off a set amount of their debt within 5 years, after which point the remaining debt is written off (The Insolvency Register, 2015).

Previous research on service encounters has examined the institutional constraints of the conversation but they have not examined how moral concerns affect the management of institutional matters. Therefore, in the first section of the analysis, we examine how concerns to avoid the negative associations of indebtedness are managed in service use encounters. In the second section, we then examine how the institutional concerns and constraints influence the delicate conversation and management of these issues.

## Method

This research is part of a series of collaborative studies with the financial advice organization which specializes in providing IVAs to individuals with substantial debts. Our data consist of 12 initial advice appointments between clients and telephone‐based advisors at the advice organization. The purpose of this advice appointment is to gather information about the client's financial circumstances and assess the viability of the client proceeding to apply for an IVA. Although it is in the company's interest for clients to enter into IVAs, it is only beneficial if the individuals are likely to meet the demands of the payment plan. In nine cases, it was the first time that the client had spoken to the advisor, and in three cases, the advisor and client had spoken briefly before but rescheduled their appointment. So as not to interfere with their decision‐making concerning the IVA, participants were not recruited until after their initial advice appointment (Speer & Stokoe, 2014). The researcher was not aware of any personal information apart from the phone number prior to the phone call and only listened to the advice recording if the client agreed to take part in the study. This procedure was approved by the ethics committee at the institution where the study took place.

Previously, the telephone advisors had followed a strict telephone script but changes over the past years have allowed for a greater deal of flexibility for the advisors. Nevertheless, there are features of the appointment which remained the same in all conversations. Typically, a conversation would start with a description of the company and the legal considerations of an IVA. The advisor and the client would then list the client's debts, income, and outgoings. Based on the client's budget and what creditors would accept, the advisor would then advise on a feasible repayment sum. An overview of alternative debt arrangements would be presented, and the clients were then invited to make a decision. The conversations differ in the narratives provided by the client and the extent to which the advisor would discuss other debt resolution options.

### Analytic method

As the advice appointments typically lasted between 30 and 90 min, there was a large amount of data within each recording and initially only eight recordings were collected. Each of the remaining four recordings was then recruited, transcribed, and a first pass of analysis was carried out independently before recruiting another recording. The sound files were transcribed using an abridged version of Jeffersonian transcription, as our primary focus was to explore how the identity of a debtor was managed (Jefferson & Lee, 1981). However, due to the element of CA in our analysis, we also transcribed short and long pauses (indicated by (.) and (…) respectively), overlapping speech (indicated by brackets) and laughter particles to improve the reading of the extracts. Advisor and client speech is indicated by ‘A’ and ‘C’, respectively. During transcription, all client, advisor, and creditor names were anonymized (as ‘Client’, ‘Advisor’ and ‘Creditor’) and numbered to differentiate between them. For example, if two client names were referred to in one advice conversation, they were transcribed as ‘Client1’ and ‘Client2’.

The initial analysis was carried out by examining the recordings for evidence of troubles‐talk or interactional difficulties in the conversation, both of which were demonstrated in Ekström *et al*.'s analysis (2013). During this process, it became evident that in these extracts debtors often gave an account of themselves which allowed them to present their identity in a specific way to avoid or manage the potentially negative associations of debt. Focusing on instances of identity management, the analysis was done inductively until no further features had been found. After twelve recordings, we could not find any further variation within these extracts of interest and we concluded that saturation had been reached (Glaser & Strauss, 1967).^1^

## Analysis

### Section 1: Managing the topic through claiming a moral disposition

One of the main problems during advice appointments is successfully managing the negative associations surrounding the topic of debt. Both parties to the conversation typically signalled their awareness of the potentially stigmatizing quality of debt on multiple occasions throughout the interview. This was evidenced by clients deploying a range of interrelated strategies to distance themselves from negative stereotypes of debtors as morally compromised or as financially irresponsible, most notably by displaying an awareness of the moral implications of their situation.

##### Responsibility: ‘We can't bury our heads anymore’

The first pattern that we identified was how a moral character can be claimed through claiming responsibility as a disposition. Ekström *et al*. (2013) had found that individuals struggling to repay their debt presented themselves as good debtors by referring to the minimization of financial problems, the reason for the problem and the role of the individual solving those problems. In our extracts, we have found other strategies which allow clients to present themselves as ‘good debtors’.

The conversation which occurs when applying for an IVA is delicate because it can be interpreted as a ‘problematic’ solution (similarly to bankruptcy) allowing individuals to write off substantial sums of debt which may have occurred in ‘irresponsible’ circumstances. This leads to situations where clients offer a moral character through claiming responsibility states or traits.

*Extract 1*

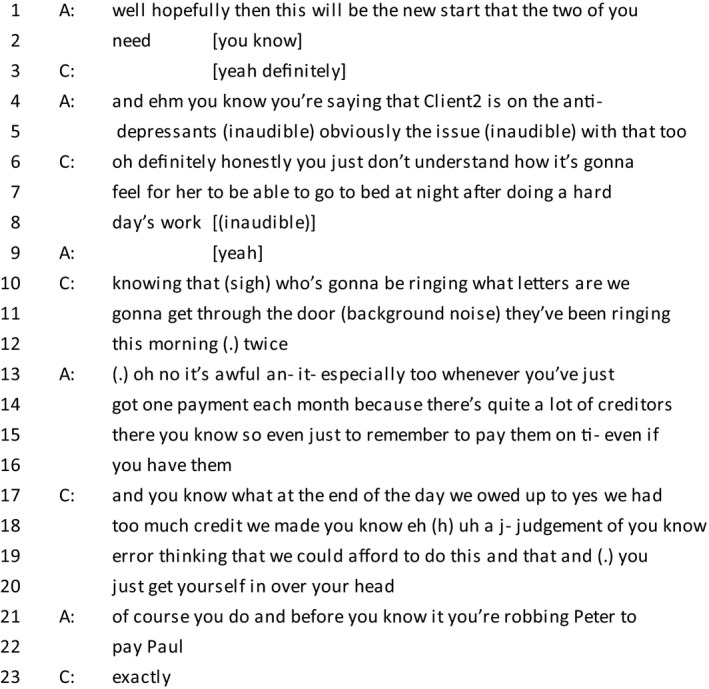

The extract above is an example of a client who gives an account of her troubles. In line 6, the client includes ‘honestly’, a phrase which often occurs when there is a confessional element that the speaker is about to disclose (Edwards & Fasulo, 2006). Towards the end of the trouble‐talk, there is a feature which is occasionally found in the transcripts whereby the client will explicitly ‘make a confession’ to present a moral character (see line 17–20). Although it may seem counter‐intuitive, a contrast is being presented between the previous actions that led to debt and the awareness of their consequences in the present. Two explanations are provided to account for why the client got into debt: Firstly, the occurrence of debt is framed as ‘a judgement of error’, a variant of the phrase ‘an error of judgement’, which is distanced from one's character. It is also prefaced with laughter, which acts as a signal of the awareness of the sensitive nature of the topic (Haakana, 2001; Jefferson, 1984). ‘Thinking that we could afford to do this and that’ implies that it was not deliberate. Both explanations are examples of causal attributions which justify how the client got into debt (Heritage, 1988). Parallels can be drawn to Ekström *et al*.'s (2013) paper, where participants used a narrative in which troubles are temporary to account for their indebtedness while preserving an identity as a responsible citizen. As in the previous example, the client marks her troubles as in the past.

In line 20, the client finishes her turn by switching footing to membership of a general category rather than speaking as an individual person (Potter & Hepburn, 2005). The switch is also evident in the advisor's response in line 21, where she refers to people in general (‘you're robbing Peter to pay Paul’) rather than the client specifically, thereby avoiding laying the blame on the client. Collaboration between the client and the advisor when building a moral account was frequently seen in these conversations, as discussed in the following section.

##### Emotion: ‘I can't stop feeling really guilty…’

The second pattern that we identified was the recurring overt display of emotion, which is traditionally seen as an uncontrollable and honest expression, reflecting an internal state (Edwards, 1999). Thus, it is unsurprising that it would occur in debt conversations (Hayes, 2000; Keene *et al*., 2015). However, in our conversations, we found that emotional displays or claiming emotion allowed the participant to claim and disclaim a number of attributes, as recognized by previous discursive research on emotion (Edwards, 1999). In these conversations, delicate moral issues were negotiated through this strategy, as evidenced below:

*Extract 2*

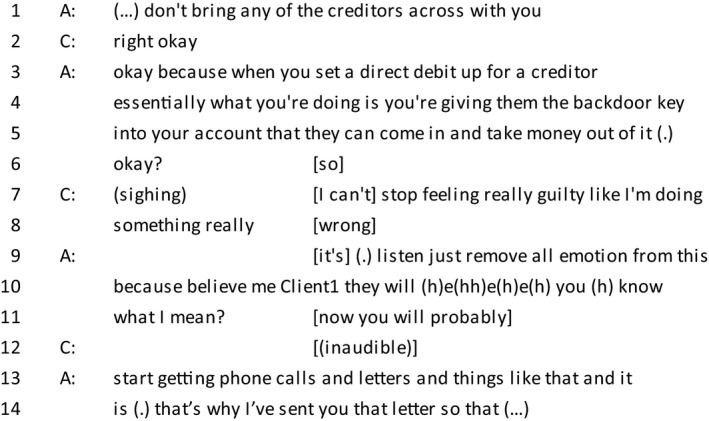

In line 7, the client begins her turn with a sigh, which has been found to work as an ‘affect forecast’ (Hoey, 2014), commonly and also in this case understood as a marker for negative affect. By positioning herself as not in control of her emotions (line 7) and then as the feelings of guilt being a consequence of the event of her ‘doing something really wrong’, she accomplishes several things: The sentence is structured as a confession which disclaims any moral irresponsibility. Further, the guilt is not presented as an internal disposition (Edwards, 1999), but rather the feeling is presented as a cognitive assessment, reflecting her knowledge of justice and fairness. This display of knowledge signals that she is aware that there is a strong contrast between her current circumstances and the ideal state of affairs and her awareness of this difference is offered as a true reflection of her character rather than the one her financial circumstances offer.

Furthermore, we can note that the participant is attending to the potentially morally problematic nature of the IVA settlement itself. Previous qualitative research has demonstrated how being in debt (Hayes, 2000) or being unable to meet repayments (Keene *et al*., 2015) can cause feelings of shame. However, in this study, the repayment option, partially writing off debt, means that an IVA could be interpreted as avoiding moral responsibility. Here, the clients’ emotional avowal of guilt is structured to counter this potential inference, being worked up through the repetitive use of ‘really’, which as an intensified phrase (Pomerantz, 1986) serves to make her statement seem more justified and genuine. This therefore serves to maintain her moral reputation while accepting an ostensibly ‘easy’ option.

The recipient of the ‘confession’, the advisor, begins her turn with an empathetic display, maintaining her role as a concerned listener who is on the client's side and providing the client with a morality account. This is carried out by contrasting the client, who is emotional and therefore a responsible character, with the creditors who are not emotional as indicated by the advisor (‘listen just remove all emotion from this because believe me Client1 they will’). Directly after ending her first turn, the advisor then makes a shift so that the institutional matters can be attended to although the client seemingly interprets ‘you know what I mean’ as the end of the turn.

##### Advisor support: ‘You've probably paid what you borrowed three times over’

In the previous examples, the client leads the moral management work, with the advisor collaborating. On other occasions, this work was initiated and led by the advisor. In contrast to other institutional settings in which troubles‐tellings are followed by minimal responses, such as between doctors and patients in Ruusuvuori's (2005) study, sometimes the advisors in this study responded emphatically to troubles‐tellings. The following extracts demonstrate patterns in which the advisor is the one who claims or collaborates in building moral dispositions on behalf of the client to manage delicate situations. An example of a delicate situation is when the client is displaying emotion, at which point the advisor would generally acknowledge the situation but ultimately needs to address the institutional concerns.

*Extract 3*

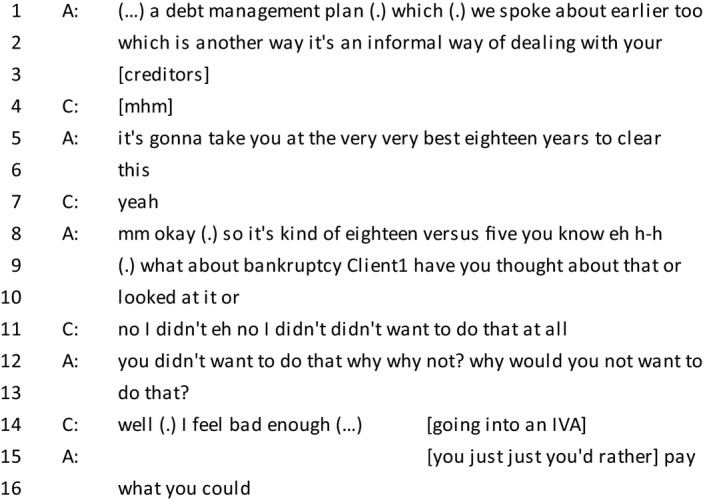

In line 2, the advisor's suggestion of bankruptcy is masked as a question, which is commonly used where explicit advising is not appropriate (Butler *et al*., 2010). As found in other examples of delicate issues (Silverman & Peräkylä, 1990), there is a pause in line 9 before approaching a delicate topic for the first time (bankruptcy) and rather than explicitly saying that bankruptcy is a viable option for the client, the advisor formulates the suggestion as a question (‘what about bankruptcy’). Indeed, the client interprets it as a suggestion rather than a question and does not wait for a marker signalling for her turn before proceeding to resist the advice. Her immediate answer could possibly be interpreted as incompetence, which may be why she follows the utterance with a repair which reframes her stance on bankruptcy as a personal choice. It can be noted that the client glosses over the word ‘bankruptcy’ by using ‘that’ instead, which has been found in other contexts of discussing delicate topics (Silverman & Peräkylä, 1990; Yu & Wu, 2015). Although the advisor persists with the line of questioning, her initial question (‘you didn't want to do that why why not’) is immediately followed by a repair that is less personal (‘why would you not want to do that’). The use of talk that is at a more general level rather than addressed to the individual is a common method of approaching delicate topics, and mitigating vocabulary is common when discussing morally sensitive issues (Linnell & Bredmar, 1996). After the advisor has persisted with the line of questioning, the client uses emotion as a resource to claim moral attributes (also seen in extract 2, ‘I can't stop feeling really guilty’). As a consequence, in line 15, the advisor abandons the line of advice and provides an interpretation of the client's feelings. This line is similar to Extract 1 in which the advisor demonstrates that she is an active listener by summarizing the client's concerns (Danby, Butler, & Emmison, 2009), but it also allows her to collaborate with the client in building a morally responsible account of her behaviour.

This active collaboration of advisors building a moral account is in stark contrast to the advisors in Ekström *et al*.'s (2013) study and more akin to the examples found in peer support hotlines (Pudlinski, 2005). Another example of collaboration can be seen in the following extract.

*Extract 4*

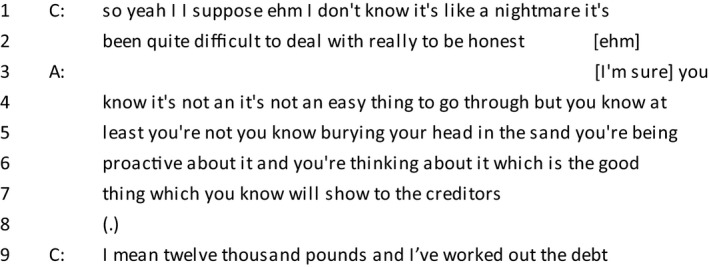

During a discussion of creditor negotiation, the client describes her situation ‘like a nightmare’, an extreme description of a negative emotion which is characterized as uncontrollable. Metaphors are commonly used to allow speakers to make use of emotion resources without having to explicitly mention them, and provide the listener with a graphic description of the speaker's circumstances (Edwards, 1999). The client continues to enforce her emotional claim and ends her utterance with ‘to be honest’, a phrase which is commonly used when offering a subjective, confessional evaluation (Edwards & Fasulo, 2006).

The advisor does interpret this as an emotional claim and starts her turn in line 3 with agreement and referring to her own expertise in the matter. This is followed by displaying a sense of understanding (Pudlinski, 2005) by offering an interpretation of the feelings of the client (Danby *et al*., 2009). This is done through attributing several positive dispositions to the client. ‘At least’, alludes to the possibility of irresponsible behaviour which the client is currently not engaging in. She finishes her turn with an incomplete sentence followed by a brief pause, accepted by the client as the end of the advisor's turn.

### Section 2: Managing institutional constraints and client resistance

In section one, we demonstrated how both the client and the advisor display awareness of the threat of the potentially stigmatizing associations of debt and successfully use various strategies to collaboratively manage these concerns. However, there are also institutional constraints on the advisor who is subject to rules set by the creditors. These often became evident towards the end of the interaction where the sum to be repaid to the creditors was calculated and the non‐negotiable details of this offer were presented to the client. At this stage, the advisor could either maintain their client‐centred focus or adopt a more overtly institutionally structured approach, both of which impacted the way that the client's moral character was managed by endorsing or undermining it.

##### Maintaining a client‐centred approach

In the following extract, the advisor has calculated a non‐negotiable repayment sum and is presenting this to the client. However, the manner in which this is done is to suggest a future line of action whilst not directly advising the client to act on the suggestion. This is commonly used in situations of advice‐giving where there is a goal to empower the clients, such as on a children's helpline (Butler *et al*., 2010). However, here this approach occasions some interactional trouble as the client construes the offer as requiring further financial concessions and a further demonstration of their moral responsibility.

*Extract 5*

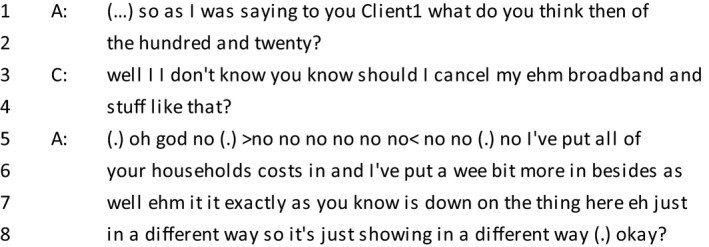

The client begins with a dis‐preferred response by avoiding an explicit rejection of the suggestion and proceeds to counter with a question (Pomerantz, 1984). The question itself pertains to the consequences of the arrangement for the client (further frugality) and serves to display their responsible consideration of these consequences. Rather than too readily accepting a debt‐reducing IVA, the client interprets the offer as an invitation to further display their acknowledgement of the implications of the arrangement.

The advisor's oh‐prefaced response signals that this is new and unexpected information (Bolden, 2006), and then proceeds with self‐repair. By making her response so extreme, the advisor both acknowledges the difficulty conveyed by the client's deliberations and reframes the issue as one of misunderstanding the detail of the calculation by referring to the budget report provided by the client. In this way, the institutional concerns of the interaction, to present and agree a repayment sum, are observed while the concerns of the client to be construed as a morally competent agent are skilfully maintained. A similar pattern can be seen in helplines where advisors can persist with advice without challenging clients’ accounts even when the advice is initially rejected (Butler *et al*., 2010).

##### Shifting the approach to expert positioning

The previous extract demonstrates how delicate negotiation can occur whilst adhering to a client‐centred approach. However, this is not always carried out by the advisor. An example is seen below after the advisor and the client have just finished the budgeting portion of the advice appointment. The client has mentioned the monthly payments that he is currently making to repay his debt. At this point, the advisor tells the client that in an IVA he would pay a substantially smaller amount than he is currently paying towards his debt and it is met with scepticism.

*Extract 6* 2

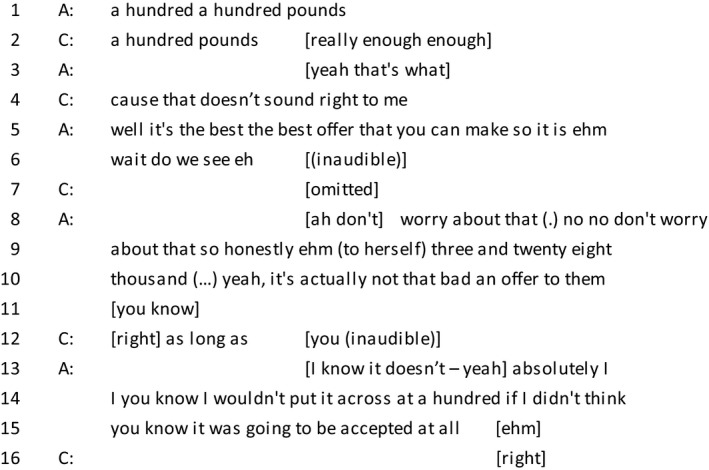

In line 4, the client resists the offer that the advisor has suggested. The statement ‘doesn't sound right to me’ works as a display of his moral character. By stating that he expected to pay more, the client is offering a responsible character in contrast to the stigma‐associated one as seen in extract 1. In contrast to the client, the advisor shifts to an expert position by stating her reply as a fact and uses an extreme case formulation (Pomerantz, 1986) to strengthen her argument (the last three words are used for emphasis.) After revisiting the numbers, the advisor softens her approach (line 8 and 10) but continues to position herself as an expert by warranting her assertion on the basis of her experience (line 14). The client accepts her offer and the conversation moves on.

At other times, the expert position is used in a way that undermines the identity of the client:

*Extract 7 (The client is listing her expenses)*

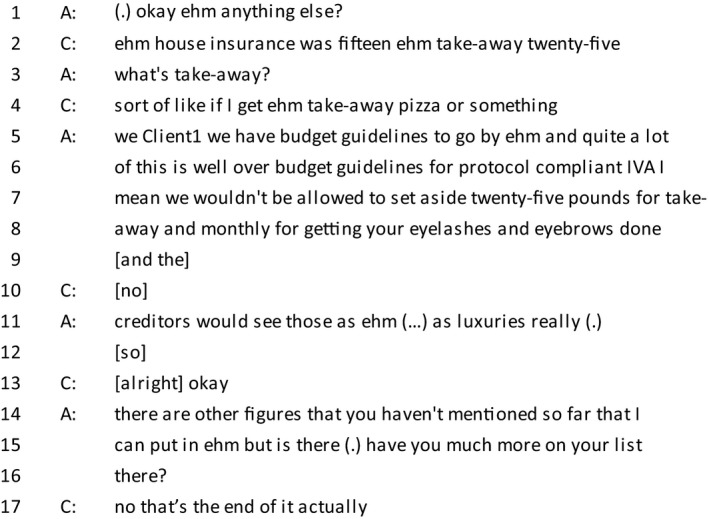

In the extract above, the advisor has just received a list of the client's expenses which are substantially higher than allowed under the guidelines for an IVA. In contrast to clients in the previous two extracts, this client does not seem to be orienting to the identity‐related concerns. This leads to the advisor using less client‐centred language than we have seen previously and although she does not explicitly reject the requested expenses, she pre‐empts advice resistance from the client and starts to build an account for her advice. By referring to herself as ‘we’, she positions herself as a category member of the company and then uses the client name, also used by counsellors in the beginning of turns that disalign with the previous turn (Butler, Danby, & Emmison, 2011). She then invokes an epistemological entitlement by speaking on behalf of the creditors and pauses, a sign of the delicate item ahead (Silverman & Peräkylä, 1990), before using the word ‘luxuries’, which is charged with negative values. This works to position the client as morally accountable for their excessive expenditure, something further reflected in the advisor's reformulation of their question on line 15 from a straightforward request for information ‘is there?’ to ‘have you much more?’, which signifies the problematic nature of further expenditure. The client orients to this undermining of her moral stance by terminating her list. While this outcome may serve the institutional demands of the encounter, this differs from previous extracts in that the advisors’ expertise has here been used to criticise rather than support the clients’ position.

## Discussion

The purpose of this study was to examine how interactants’ negotiation of the potentially stigmatizing associations of debt might affect debt advice conversations. We found that both the advisor and the client managed these negative associations through disclaiming the stigmatized identity associated with debt and that the advisor would typically use a client‐centred approach, allowing the client to privilege their own account of their situation. However, in order to service the institutional goals, the advisor would occasionally shift their positioning to that of expert. On occasions, this was evidently problematic as it could undermine the face‐saving strategies of clients.

At the outset, this paper adds to the current body of literature on advice conversations from a discursive perspective. While authors such as Linnell and Bredmar (1996) have identified strategies used to manage sensitive topics in service use interactions, and previous research on institutional talk has examined expert or client‐centred talk (Butler *et al*., 2010; Emmison *et al*., 2011; Maynard, 1991; Peräkylä, 1993), no research has examined these features of advice‐giving in tandem. Our research illustrates how institutional constraints can serve to undermine the delicate face‐saving collaboration between service provider and user, through shifting their interactional dynamics. We suggest that further research is required into how the changing policy frameworks of statutory and private services operate to structure their institutional requirements and thereby serve to counter or reproduce stigma in service use encounters.

A further set of findings pertain to the multiple epistemologies attended to by participants. On one hand, the advisor has the role of expert, from which multiple resources can be drawn. They have access to training and documents on IVAs, experience of advising previous clients and a unique relationship with creditors, all of which can strengthen or discount clients’ accounts. On the other hand, the client has access to their personal experiences and knowledge of their current circumstances which is also vital to the success of the interaction, but which is fraught with stigma‐management concerns. During the appointment, the advisor can therefore pursue one of two strategies, using a client‐centred approach or adopting an expert footing. Where the client is treated as the expert upon their own circumstances, this typically serves to elicit accurate data, necessary for the success of the service encounter. When this diverges from the institutional constraints of the conversation, an expert positioning can enable the advisor to redirect the interaction towards institutional goals. However, if this shift in footing undermines clients’ concerns, it can make the negative associations of debt explicit and unavoidable for the client and also undermine their entitlement to speak. Although in our data the advisors treated these positions as discrete, a further practical implication is therefore that interactional strategies which manage both the institutional goals and client concerns should work best to keep the client engaged.

Our third contribution builds upon previous research into the specific dynamics of debt advice which has primarily examined the initial barriers to seeking advice (Dearden *et al*., 2010; Goode & Waring, 2011; Pleasance *et al*., 2007). Our research extends this research by examining actual service use encounters, finding that even when individuals overcome these initial barriers, debt remains a sensitive topic (Hayes, 2000). To negotiate this, clients can use several interactional strategies to disclaim the identity associated with debt and reclaim a moral character, including through using emotional discourse and by explicitly claiming responsibility. Both these strategies signal awareness and disapproval of the opposing, undesirable character (i.e., a financially irresponsible and immoral character) thereby serving to signal an opposing moral position.

However, these strategies were found to be problematized by the nature of the advice on offer. Although one advantage of IVAs is the opportunity to discharge some of the debt, we found that this aspect of the solution was seen as problematic for some clients: the implications of defaulting on debt and not paying back the full amount required clients to perform additional identity management to demonstrate that it was not considered the ‘easy way out’. In order to remedy this problem, the advisor typically collaborated with the client in building a moral account to manage delicate circumstances in the conversations, often through empathy but also through invoking their own expertise in the area. Hence, one practical implication of our findings is that debt advice agencies need to consider how the solutions they offer may ironically reproduce the stigma felt by potential clients. The repackaging of products as a ‘morally responsible’ choice for themselves, their families, and their creditors could afford an effective face‐saving strategy that enables more effective service uptake.

Finally, our research also addresses the broader literature on stigma and the issue of service uptake among potentially stigmatized groups. Previous research has examined retrospective accounts of stigmatized service use leading to disengagement from community and social services (Campbell & McLean, 2002; McLean *et al*., 2003; Stevenson *et al*., 2014). These studies found that the expectation and experience of prejudice worked as a barrier to future service engagement. In contrast to this previous research, our study found that debt advisors often undertake complex collaborative work to enable clients to save face within this encounter, though this may be constrained by institutional requirements. A final conclusion then is that the manifestation and management of stigma in service use is a more complex and multifaceted phenomenon than previously considered, and that stigma can be considered as a collaborative outcome of institutional talk where both participants manage moral accountability concerns.

As our study is on a small and selective sample of advice appointments, it is unlikely to span the entire range of possible debt advice interactions in the United Kingdom much less those in countries with different levels of debt and debt advice provision. Moreover, the advisors in the current study belong to a private company and the advice appointment has a specific goal of assessing how appropriate legal debt restructuring is for the client. In contrast, other debt advice agencies may focus on more practical concerns, for example budget management, which may lend themselves to different stigma‐management and epistemological concerns. Regardless of the type of debt advice that is offered though, we argue that the conversation is likely to be difficult due to the difficulties associated with debt (Hayes, 2000). By examining the unfolding of advice conversations, we can see how interactional strategies have an immediate effect on the conversation. However, as our research considers only the initial encounters without examining the subsequent stages in the debt management process, we propose that future research examines the link between the content of the conversation and debt advice outcomes. By taking this approach, we would be able to examine the relationship between the interactional strategies used in the initial debt advice appointment and engagement with the debt resolution process, which is beyond the scope of the current study. In doing so, we can begin to better understand the link between these micro‐processes of service use and their wider personal and social consequences as well as how to design more engaging and more effective service provision for vulnerable social groups.

## Notes

1 Although the concept of data saturation originates from the grounded theory methodology (Glaser & Strauss, 1967), it was used as a guideline during data collection.

2 The line omitted is the client talking primarily to himself about creditors. Although it is an interesting feature, it was omitted for readability and due to not being directly relevant to the rest of the extract. The next turn made by the advisor, in line 8, references the client's previous concerns in line 4.
